# Oculomotor Vergence Eye Movement Endurance in Normal Vision via Virtual Reality-Integrated Eye Tracking

**DOI:** 10.3390/jemr19030049

**Published:** 2026-05-05

**Authors:** Fatema F. Hirani, Farzin Hajebrahimi, Tara L. Alvarez

**Affiliations:** Department of Biomedical Engineering, New Jersey Institute of Technology, Newark, NJ 07102, USA; ffh@njit.edu (F.F.H.); farzin.hajebrahimi@njit.edu (F.H.)

**Keywords:** vergence eye movement, convergence, divergence, virtual reality, vergence endurance

## Abstract

Modern societies are becoming increasingly dependent on electronics, leading to an increase in visual symptoms. Vergence endurance, the ability to sustain performance, may serve as a quantitative metric to complement symptom surveys to assess vergence performance during near visual tasks. To quantify vergence endurance, 48 participants, aged 15 to 23 years with normal binocular vision, completed a 15 min symmetrical disparity vergence step task to assess potential changes in peak vergence speed over the course of the experiment. Peak velocity, final amplitude, and the slope of the linear regression fit of the peak velocity as a function of stimulus recording were quantified for convergence and divergence responses using an eye tracker integrated in a virtual reality headset. Peak velocity was sustained by 63% and 69% of participants for convergence and divergence eye movements, respectively. Convergence and divergence responses were significantly different for peak velocity (*p* < 0.001) and vergence endurance (*p* < 0.03). The endurance metric tool has potential that may help shape future clinical applications for those with acquired brain injuries, including concussions or neurodegenerative diseases such as multiple sclerosis or Parkinson’s disease.

## 1. Introduction

Binocular coordination is the process by which the extraocular muscles rotate the ocular globe so that the object of interest is projected to the fovea, the area of the retina that contains the highest density of photoreceptors. Vergence eye movements are the inward or outward rotation of both eyes using disconjugate movement. Those with normal binocular vision typically move their eyes effortlessly and experience no visual symptoms throughout the day. Yet, handheld devices and digital screens are increasingly becoming part of modern society [1]. Visual fatigue is commonly reported after prolonged use of digital devices [2]. Extended use of digital devices is increasing visual symptoms, even among those with normal binocular vision, leading to complications such as computer vision syndrome (CVS) [3]. Recent reports indicate that CVS, also called digital eye strain (DES), is becoming more prevalent, affecting about 70% of the population due to increased screen time [1,3,4]. Visual symptoms assessed with subjective visual symptom surveys support the association between sustained computer use and visual symptoms [5,6]. However, subjective visual symptom surveys may lack specificity and/or sensitivity compared to the objective assessments [7,8,9]. A review concludes that DES is commonly observed in those with accommodative and vergence dysfunction [10]. Further research is warranted to determine whether a potential link exists between vergence dysfunction and DES, and, if so, to identify the underlying mechanism. Hence, designing an objective protocol to assess repetitive vergence endurance may have greater clinical utility than questionnaires for assessing visual symptoms in both patient and healthy populations. These designs would be critically beneficial for various patient populations in which binocular vision dysfunctions associated with eyestrain and visual fatigue are common.

Repetitive eye movements occur throughout the day and have been used to assess fatigue in patient populations. Specifically, the gradual reduction in peak velocity in saccades, the conjugate side-to-side eye movements used during reading, is suggested as an indicator of fatigue in patients with multiple sclerosis assessed via a 2D screen [11]. Studies focused on vergence eye movements in clinical populations have shown that patients with brain injury have a high prevalence of convergence dysfunctions [12,13,14], as do patients with neurodegenerative diseases such as Parkinson’s disease [15,16] and multiple sclerosis [17]. Similarly, a pilot study supports that the speed of vergence eye movements is also reduced after prolonged repetition in those with a diagnosed concussion assessed via a haploscope [18]. A current gap in the literature is the assessment of vergence eye-movement performance while using a virtual-reality headset. This study aims to develop a practical 15 min test that is feasible for clinical use to assess vergence endurance in future patient populations. The novelty of this study is the use of the vergence system using symmetrical step stimuli in a virtual reality headset, which has the potential to be easily translated to the clinic. The peak velocity of vergence step responses is chosen because it is easily measured quantitatively and, when reduced, indicates that the vergence system will take a longer time to fuse a target. Our system will measure vergence peak velocity throughout the test and calculate the slope of a linear regression of the peak vergence velocity as a function of response number, which is used to quantitatively measure the vergence system performance, defined as vergence endurance.

Before patient studies of vergence endurance commence, this phenomenon must be assessed in individuals with normal binocular vision to serve as a comparison and determine whether endurance is affected by neural damage, dysfunction, or disease. Hence, the present study will assess vergence endurance, defined as the ability to sustain vergence eye movement performance over time. Endurance will be quantified by assessing potential changes in peak velocity during a repetitive vergence eye-movement task in individuals with binocularly normal vision. This study tests the primary hypothesis that the vast majority of participants with normal binocular vision will maintain sustained convergence and divergence oculomotor performance during a 15 min binocular eye-movement test defined as a consistent peak velocity throughout the test, whereas some individuals may show a decrease in performance defined as an observed decrease in peak velocity as the test progresses. A secondary hypothesis to be tested is that differences will be observed in convergence and divergence performance because each system has unique cells. Since the divergence system has reported fewer velocity-encoding cells in the midbrain compared to the convergence system [19], the secondary hypothesis tested is that the divergence system performance will not be maintained as well as the convergence system. The findings of this study may lay the foundation for future clinical studies to quantitatively assess vergence endurance during a repetitive eye-movement task across various patient populations, which is beyond the scope of this study.

## 2. Materials and Methods

### 2.1. Participants

Prior to enrollment, adult participants provided written informed consent. For minors, the legal guardian provided written consent, and the minor provided written assent. The written informed consent and assent were approved by the New Jersey Institute of Technology Institutional Review Board, for the ethical review of the experiment in accordance with the Declaration of Helsinki.

### 2.2. Eligibility Criteria

Ethics approval enabled the study to enroll participants aged 11 to 25 years, recruited from surrounding communities. The age range of those who enrolled was 15 to 23 years. This age range was chosen to ensure they could follow directions, were not presbyopic, and had a developed visual system. To be included in the study, participants had to have normal binocular vision and be asymptomatic. The criteria for normal vision were defined as refractive correction, if needed, to achieve a visual acuity of 20/25 or better in each eye; normal local stereopsis of 70 arc-s or better; normal global stereopsis of 500 arc-s or better; and a normal optometric sensory–motor exam as described below.

Individuals with hyperopia > 5 diopters (D), myopia > 6 D, anisometropia > 1.5 D, vertical heterophoria ≥ 2 prism diopters (Δ), astigmatism > 4 D, and any unwillingness to utilize prescribed refractive correction were excluded from the cohort. Other exclusion factors were the presence of manifest or latent nystagmus, a history of prior strabismus, intraocular, or refractive surgeries, or any disease affecting accommodation, vergence, or ocular motility, such as Graves’ disease, Parkinson’s disease, myasthenia gravis, or multiple sclerosis, having a previous history of mild-traumatic brain injury (mTBI), diagnosed concussions, constant strabismus at-near or at-far, attentional disorders, or prior vision dysfunctions.

### 2.3. Optometric Sensory–Motor Examination

The optometric sensory–motor exam followed the same protocol as many randomized clinical trials and included near point of convergence, positive and negative fusional vergence, near and far heterophoria, vergence facility, monocular amplitude of accommodation, and accommodative facility [20,21,22,23]. The near point of convergence was measured along the midline from the bridge of the nose using a 20/30 equivalent column of letters at 40 cm and a near point rule. The distance at which a participant reported diplopia was the breakpoint, measured in cm, as shown in our instructional video [24]. The target was then receded by moving it away from the participant along the midline. When the participant regained fusion, the near-point of convergence recovery was recorded in cm. Normal near point of convergence break is defined as less than 6 cm [25,26,27,28]. The cover–uncover test was used to objectively measure dissociated phoria with a prism and an alternate cover test at near (40 cm) and far (6 m). Negative and positive fusional vergence were measured with a prism bar (1Δ, 2 to 20Δ in 2Δ increments, 20 to 45Δ in 5Δ increments). Prisms were changed at a rate of 2Δ/s while looking at a 20/30 equivalent column of letters located at 40 cm. Positive and negative fusional vergence were recorded as the point when the visual target had sustained blur or became diplopic if blur was not perceived. Normal ranges were defined as passing Sheard’s criteria [29] with a minimum positive fusional vergence of 15Δ. The vergence facility was assessed with a 12 base out (BO) and 3 base in (BI) Δ prism set using a 20/30 equivalent column of letters at a measured distance of 40 cm, where the number of times the participants could see the visual target clear and fused was counted and recorded as cycles per minute (cpm). The procedure was repeated at a distance of 6 m.

Accommodation was assessed using monocular amplitude of accommodation for the right eye and repeated for the left eye using the Astron Accommodative Rule. A vertical column of 20/30 letters placed at 40 cm was slowly moved towards the participant until they reported blur, as shown in our instructional video [24]. Hofstetter’s rule of 15D–¼ * age was used to determine normalcy [30]. Accommodative facility was measured with a +2/−2D flipper lens using the right eye with the left eye occluded. The number of times a participant could perceive clear vision while viewing a 20/30 vertical column placed at 40 cm along midline with a +2D lens and then with a −2D lens was recorded in cpm.

Visual symptoms were assessed using the Convergence Insufficiency Symptom Survey (CISS), a validated 15-question instrument with a 4-point Likert scale ranging from 0 (symptoms never occur) to 4 (symptoms always occur), yielding a score of 0 to 60 points. [31]. The symptomatic cutoff was 21 points or greater for adults and 16 points or greater for children, as used in prior studies [20,21,22,23,31]. The CISS was used only to ensure that participants were asymptomatic, which was part of the study’s inclusion criteria.

### 2.4. Objective Eye Movement Recordings

**Figure 1:**
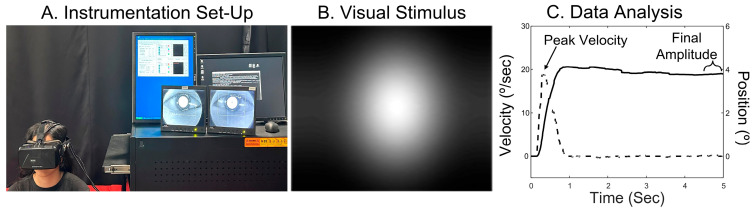
Data acquisition and analysis. (**A**). Instrumentation set-up. (**B**). Visual stimulus. (**C**). Data analysis of a typical eye movement response showing binocular position (solid line) and velocity (dashed line) to assess peak velocity (shown by arrow) and the averaged last half second of position to assess final amplitude (shown by curly bracket).

Objective eye movement recordings were quantified with an ISCAN infrared (λ = 940 nm) video-based eye tracker (Woburn, MA, USA) that has a sampling rate of 240 Hz (temporal resolution of 4 ms) and a spatial resolution of 0.1° integrated into an Oculus DK2 virtual reality headset (Oculus, Meta Platforms Inc., Menlo Park, CA, USA), shown in Figure 1A. The Oculus DK2 uses a 14.5 cm display with a 960 × 1080-pixel resolution per eye, a 93 degree field of view, and a 75 Hz refresh rate. To maintain hygiene, disposable face masks were provided for use with the virtual reality (VR) headset. Participants placed the VR headset, then tightened the strap to reduce potential drift in the recordings. Once the headset was comfortable, the participant was asked not to adjust the headset during the test and to sit still in a comfortable chair. One benefit of a head-mounted VR system compared to a table-mounted eye-movement tracking system is that, if the participant moves their head slightly, the eye-tracking system moves with them. Hence, there is less drift in the responses. The visual stimulus was a distribution of Gaussian that had a 2° eccentricity to primarily stimulate retinal disparity and minimize retinal blur, as shown in Figure 1B [32].

Eye movements were monocularly calibrated at the beginning of the session using targets at 1°, 3°, 5°, and 7° inward rotation for the right eye, and then repeated for the left eye. These known vergence demands were verified using an external calibration stimulus display, in which we fixed the headset and ensured that the stimuli on the screens within the head-mounted display aligned with a known physical distance in real space. Monocular calibration was chosen to reduce the influence of fixation disparity on calibration curves [33]. There was a pause between each 1 s calibration measurement recorded. Auditory cues with verbal instructions helped to reduce the occurrence of blinks during the calibration procedure. While vergence steps, vergence pursuits (ramps), and saccades were recorded to reduce prediction, the vergence endurance protocol analyzed responses to 4° symmetrical disparity stimuli for both convergent and divergent responses. Convergent responses began at a 4° vergence demand and finished at an 8° vergence demand, and vice versa for divergence responses. Before each change in vergence demand, a random delay of 0.5 to 2.0 s was introduced to reduce predictive anticipatory movements [34]. In total, 35 convergence and 35 divergence movements were collected and analyzed. The 4° symmetrical step change in vergence was used because it yields a good signal-to-noise ratio while also not being too large in disparity to elicit many saccadic intrusions [35,36,37].

### 2.5. Eye Movement Analysis

All objective eye-movement data were analyzed using a custom MATLAB software package Version 2023a [38,39]. Eye-movement responses were filtered with a 4th-order Butterworth low-pass filter with a cutoff frequency of 40 Hz [21,40,41,42,43]. Individual participant calibration curves were calculated using linear regression to convert acquired voltage values to degrees of ocular rotation. Responses were then monocularly calibrated. Individual responses were subtracted, where convergence eye movements were plotted as positive, and divergence eye movements were plotted as negative. The final amplitude was measured as the average of the last 0.5 s of the position responses, as shown in Figure 1C (curly bracket). The derivative of the eye movement position response was calculated using a two-point central difference algorithm to assess the peak velocity of each individual eye movement response, as shown in Figure 1C (arrow). Blinks were identified by a saturated signal in the eye-movement position trace; if the blink occurred during steady state, the signal saturation was removed, and the eye movement was still analyzed. Participants were instructed not to blink when the stimulus abruptly changed to prevent a blink from masking the peak velocity. Blinks during the transient portion were omitted from the analysis, which rarely occurred in these datasets.

Endurance was evaluated by plotting each convergent response peak velocity as a function of the stimulus number within the oculomotor test. A linear regression was performed to determine the slope of any potential trend in peak velocity. Specifically, a positive slope indicated an improvement in performance, a negative slope indicated a decline in performance, and a slope close to zero indicated no discernible change or stable performance. This analysis was repeated for divergent eye movements. This was repeated for each divergent response peak velocity. The slope was assessed for each individual participant and then pooled for group-level analysis. Prior research on four participants with normal binocular vision conducted a much longer experiment, either 100 vergence steps or 1000 saccades, and reported a 20% decrease in peak velocity from the end to the beginning of the experiment [44]. Since vergence endurance is a new metric, we used this prior study that defined a 20% decrease in performance as a substantial reduction, since their test included about three times as many observations. Post hoc, the average peak velocities for convergence and divergence observed in our 48 participants were approximately 20°/s and 15°/s, respectively. Using the prior study, a 20% reduction would yield an end-of-test peak velocity of about 16°/s for convergence responses and 12°/s for divergence responses across the 35 movements observed. This equates to linear regression slopes of 0.11 and 0.09 for convergence and divergence responses, respectively, which we averaged to 0.1. Hence, we defined stable performance as a slope from the linear regression fit of ±0.1. An improvement in peak velocity was a linear regression slope > 0.1. A degradation in performance was defined as a slope < −0.1. The percentage of participant responses within each category (stable, improvement, and degradation) was calculated for convergence, and the same calculation was repeated for divergence responses.

### 2.6. Statistical Analysis

Linear regressions for all the endurance responses were calculated using a custom MATLAB code. Vergence endurance was defined as the slope of the linear regression, assessed for each participant for convergence and then repeated for divergence. The average, standard deviation, 95% confidence intervals (95% CI) for peak velocity, final amplitude, and vergence endurance were calculated for the 4° symmetrical disparity step responses to convergence and divergence using Jamovi (Version 2.7.13). A Shapiro–Wilk test was performed to assess the data’s normality. A paired *t*-test was performed on the following convergence and divergence metrics: peak velocity, final amplitude, and vergence endurance (slope of linear regression analysis), assuming normal data distributions.

To summarize, the data analysis pipeline/scheme consisted of the following: (1) calibrate responses, (2) filter responses, (3) measure peak velocity and response amplitude per response for each participant, (4) aggregate the peak velocity as a function of stimulus response number during the test, (5) calculate the slope of the linear regression, and (6) compute group-level statistical analysis.

## 3. Results

### 3.1. Participant Demographics and Optometric Exam

Participants were equally distributed by sex, and their ages ranged from 15 to 23 years, with a mean and standard deviation of 18.8 ± 1.8 years (95% CI: 18.3 to 19.3 years). Of the 48 participants, 50% were female, and 50% were male. The majority of participants were non-Hispanic (87.5%). For race, 48% were Caucasian, 35% were Asian, 10% were Black, 4% did not wish to report race, and 2% listed more than one race. Table 1 summarizes sensory–motor information, including symptom scores, and confirms that participants had normal binocular vision and were asymptomatic. All participants were asked verbally about their experience during the 15 min virtual reality oculomotor test. None of the participants reported cybersickness, visual fatigue, eye strain, or eye dryness. Some participants (*N* = 8) reported discomfort with the head-mounted display, which added pressure to the top of their nose.

### 3.2. Typical Behavioral Data

**Figure 2:**
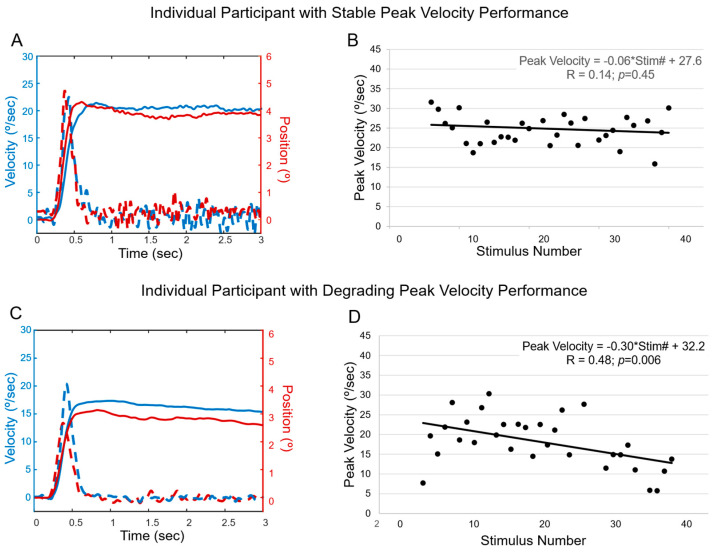
Eye movement data and individual-level analysis of the two most common behaviors observed. The data represent two sample participants. (**A**) Individual participant responses who maintained vergence performance (stable performance) from a 4° symmetrical vergence step stimulus for average position (solid line) and average peak velocity (dashed line) for the first ten responses (blue lines) and the last 10 responses (red lines). (**B**) Linear regression of convergence peak velocity as a function of stimulus number. Each dot represents a single observation. Stim# is the stimulus number (**C**) Same nomenclature as plot 2A of a participant who decreased performance when comparing the peak velocity and position for the first compared to the last ten responses. (**D**) Linear regression of a participant whose vergence performance decreases as the experiment progresses.

Two individual participants are plotted in Figure 2, showing typical behaviors from this population sample. Figure 2A,B depict an individual who maintains oculomotor performance across a series of 35 convergence movements, whereas Figure 2C,D show an individual with a decrease in oculomotor performance. Figure 2A plots that the average first 10 (blue lines) and last 10 (red lines) position (solid line) and velocity (dashed line) traces for the convergence tasks are relatively similar. Conversely, Figure 2C plots that the last 10 position and velocity traces for the convergence tasks have lower peak vergence velocity and final amplitude than the average first 10 responses. This is similar to the data shown in Figure 2B,D, where peak vergence velocity for the 4° convergence task is plotted as a function of stimulus number. Figure 2B has a relatively flat line with a slope of −0.06 from the linear regression, indicating that the convergence peak velocity did not change significantly during the experiment (*p* = 0.5). Figure 2D has a negative slope of −0.3 (*p* = 0.006), indicating that the change in vergence peak velocity differs significantly with stimulus number. In other words, as the experiment progresses, the peak velocity significantly decreases.

### 3.3. Group-Level Data Analysis

**Figure 3:**
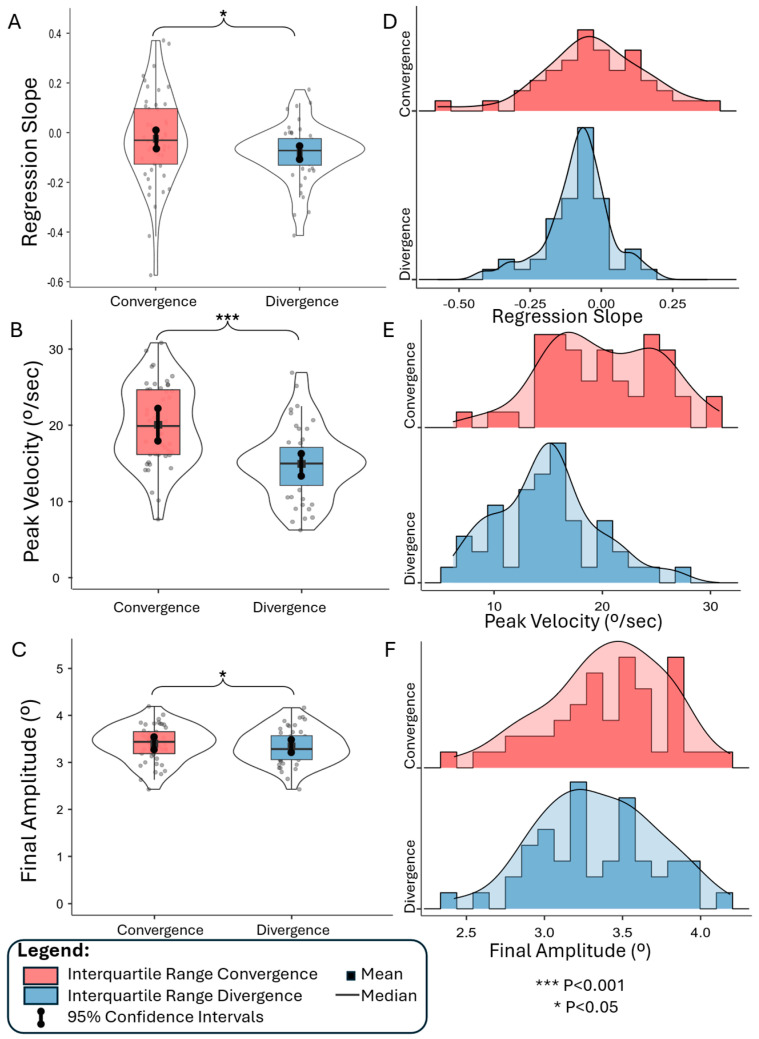
Group-level results. Each circle is a single observation. Violin plots of linear regression slope (**A**), peak vergence velocity (°/s) (**B**), and final amplitude (°) (**C**). Histogram plots of regression slope (**D**), peak vergence velocity (°/s) (**E**), and final amplitude (°) (**F**). Convergence is plotted in red and divergence in blue.

The group-level results are shown in Figure 3 as violin plots showing the mean (solid line), median (square box), interquartile ranges (red box for convergence and blue box for divergence), the 95% confidence intervals (CI) as vertical lines, and distribution of the samples for the slope of the linear regression (3A), peak velocity (3B), and final amplitude (3C). Endurance is evaluated using the slope of the linear regression displayed in Figure 3A. The mean linear regression slope for the convergence task was −0.03 (95% CI: −0.08 to 0.03), and −0.08 (95% CI: −0.11 to −0.05) for the divergence task. The range for the convergence linear regression slopes was −0.57 to 0.37, while the range for the divergence linear regression slopes was −0.41 to 0.17. Results are summarized in Table 2. Using a prior definition for stable, improved, or degraded performance explained within the methodology, our results show that for convergence movements, 30/48 (63%) participants had stable performance, 11/48 (23%) participants showed decreasing convergence peak velocity or performance degradation, and 8/48 (17%) participants exhibited increasing convergence peak velocity or improved performance as the experiment progressed. Using the same criteria for divergence movements, 33/48 (69%) participants maintained stable divergence performance, 14/48 (29%) showed a decrease in divergence peak velocity, and only 1/48 (2%) showed an increase in divergence peak velocity as the experiment progressed. The datasets were normally distributed, as indicated by a Shapiro–Wilk normality test (*p* > 0.05). Using a paired T-test, significant differences were observed between the endurance of convergence and divergence responses [T(47) = 2.21; *p* < 0.03], with a small-to-moderate Cohen’s D effect size of 0.32. Convergence responses showed more consistent peak velocity performance throughout the experiment than divergence responses.

Figure 3B summarizes the group-level peak vergence velocities, where the convergence mean was 20.0°/s (95% CI: 18.5 to 21.6°/s), and the divergence mean was 14.9°/s (95% CI: 13.6 to 16.2°/s). The range for convergence peak velocity was 7.6 to 30.8°/s, and for divergence peak velocity was 6.2 to 26.9°/s. The Shapiro–Wilk normality test also showed that the peak velocity datasets were normally distributed (*p* > 0.05) and significantly different [T(47) = 6.37; *p* < 0.001], with a large Cohen’s D effect size of 0.92. The convergence peak velocity of responses was faster than the divergence responses. Group-level final amplitude is summarized in Figure 3C, where the mean convergence final amplitude was 3.4° (95% CI: 3.3 to 3.5°) while divergence final amplitude was 3.3° (95% CI: 3.2 to 3.5) with a difference between averages of 0.06°. An unpaired *t*-test showed significant differences were observed between the final amplitude of convergence and divergence responses [T(47) = 2.22; *p* < 0.03], with a small-to-moderate Cohen’s D effect size of 0.31. The resolution of our eye tracker was 0.1°; hence, the difference in final amplitude between convergence and divergence responses should be interpreted with caution.

Histograms of vergence endurance (slope of the linear regression), peak velocity, and final amplitude for convergence (red) and divergence (blue) are shown in plots 3D, 3E, and 3F, respectively. Data are also summarized in Table 2 for convergence and divergence responses for the slope of the linear regression, peak velocity, and final amplitude.

## 4. Discussion

Studying participants with binocularly normal vision showed that, for both convergence and divergence movements, the majority of participants maintained stable performance during a 15 min oculomotor test, as indicated by a sustained peak velocity throughout the experiment, assessed by the slope of a linear regression fit. Furthermore, significant differences were observed between the convergence and divergence eye-movement responses, providing further support for the oculomotor field: although both are disconjugate movements, they constitute distinct systems.

### 4.1. Vergence Endurance Test

Computer vision syndrome (CVS), also known as digital eye strain (DES), is becoming more prevalent as digital devices are increasingly integrated into daily life. CVS is prevalent in 16% of children [45] and 50% to 77% of adults [46,47,48,49]. It is typically assessed via symptom surveys. Reviews and meta-analyses of CVS conclude that CVS is commonly associated with the following visual symptoms: eyestrain, double/blurred vision, eye irritation, and redness [50,51,52]. Furthermore, a meta-analysis concludes that the physiological mechanism underlying CVS remains poorly understood [51]. Recent papers support the idea that tear-molecule concentrations may serve as biomarkers for CVS/DES [53]. While other studies show that binocular and accommodative dysfunctions have a high co-morbidity with CVS [54,55,56], especially with the increased accessibility of virtual reality headsets [57]. While reviews include remediations such as visual rehabilitation, visual breaks, and ergonomic changes in the workforce, quantitative methods to assess their effectiveness are scarce. Taking visual breaks to reduce visual symptoms of CVS supports the notion that the ability to maintain binocular vision is compromised by prolonged visually demanding work, especially when using digital devices. Perhaps vergence endurance is worse among those with CVS than among those without visual symptoms. Quantitative methods in conjunction with symptomology assessments would reduce bias and subjectivity. Given the high comorbidity between binocular dysfunctions and CVS, objective eye-tracking, particularly of vergence eye movements, may help assess treatment effectiveness.

Prior literature has quantitatively investigated the ability to maintain oculomotor performance in those with normal binocular vision. Yuan and Semmlow (2000) systematically investigated 4° symmetrical convergence responses before and after repetitive convergence steps, convergence ramps, and saccadic movements within a haploscope [44]. For their experiment with only convergence steps, they report a 20% decrease in convergence peak velocity for the mean of the last 50 convergence steps compared to the initial 50 convergence steps, with 100 convergence steps in between, for a total of 200 convergence-step stimuli [44]. Their experiment was the model for our current experiment, in which we selected far fewer stimuli to create an oculomotor test suitable for future clinical testing across various patient populations. Other clinical studies have investigated repetitive eye-movement tests to quantitatively assess symptoms of visual fatigue. Multiple sclerosis patients with internuclear ophthalmoparesis due to demyelination of the medial longitudinal fasciculus were studied using a repetitive saccade test and compared with healthy participants to quantitatively assess visual fatigue reported in this patient population using a 2D visual stimulus monitor [11]. They report that 75% of patients show a decrease in saccadic peak velocity, whereas healthy participants show no significant change in saccadic peak velocity during the oculomotor test. A review of several neurodegenerative diseases also reports decrements in saccadic performance and recommends objective eye-movement recording to assess underlying neural degeneration [58]. Acquired brain injury, specifically concussion patients, also routinely report visual fatigue [13]. One oculomotor test used distractors, presented with vergence eye-movement stimuli, to assess performance. Vergence responses showed a degradation in accuracy, and precision was observed in those with a concussion but not in healthy individuals; yet, this task was challenging for many concussion participants [18]. Hence, the present task builds on prior studies in both patient populations and healthy individuals that use an oculomotor test to quantitatively assess potential performance degradation that patients can feasibly complete within a virtual reality head-mounted display with integrated eye trackers.

While most participants showed stable performance, 17% of participants and 2% of participants for convergence and divergence, respectively, increased their peak velocity as the 15 min test progressed. The increase in peak velocity is speculated to be due to adaptation or sequential learning of the protocol. Interestingly, the results showed that more participants increased their peak velocity during convergence than during divergence responses. The underlying neurophysiology shows that, in macaques, more velocity-encoding convergence burst cells were found in an area of the mesencephalic reticular formation just dorsal and lateral to the oculomotor nucleus than divergence burst cells [19,59]. Furthermore, convergence burst cells were observed in the more dorsal mesencephalic region, rostral to the superior colliculus, where divergence burst cells were not described. Perhaps synchronization of the burst cell firing rates leads to a higher overall peak velocity. Since primate studies support the presence of more convergence than divergence cells, one can speculate that the difference in the number of convergence-to-divergence burst cells may, in part, explain the observation that more participants show an increase in peak velocity during convergence responses, compared to peak velocity in divergence responses. On the contrary, more participants exhibited reduced performance in the divergence system (29%) than in the convergence system (23%). Prior research investigating repetitive vergence eye movements speculates that the divergence eye movement system may be more vulnerable to fatigue than the convergence eye movement system, and may, in part, result from saturation of the divergence pulse, leading to reduced performance in peak speed and accuracy [44]. More research is warranted to determine the underlying mechanism that accounts for the difference between the convergence and divergence eye-movement systems. One potential explanation may lie in electrophysiological studies in nonhuman primates.

### 4.2. Potential Underlying Neurophysiology

Abrupt changes from one vergence demand to another, a vergence step response, have been explained using the Dual Mode Model [36,43,60,61]. In a vergence step eye movement, a preprogrammed fusion-initiating component (FIC) triggers eye rotation, leading to a rapid decrease in retinal disparity, describing the system’s speed. In addition, a feedback-controlled fusion-sustaining component (FSC) is used to fine-tune the system, further reducing retinal disparity so that the object of interest is projected to the fovea, describing the system’s accuracy. The Dual Mode Model is based on neurophysiology research, in which the FIC models the ‘velocity-encoding’ burst or phasic cells recorded in the midbrain’s supraocular area [19,62]. The FSC is based on the ‘position-encoding’ or tonic cells, which are in a similar vicinity [59]. The FIC is responsible for vergence eye movement speed, while the FSC is responsible for response accuracy. Specifically, the peak velocity of a vergence response is driven by the FIC, the velocity-encoding burst cells. Hence, the vergence endurance test developed here assesses whether the FIC is changing and, if so, how it is changing. Our results showed that, for over 60% of participants with normal binocular vision, the peak velocity of either convergence or divergence responses remained stable during the vergence endurance test. Hence, we speculate that for most participants, the FIC within the supraocular area may have stable performance. We observed that about a third of the participants showed a decrease in peak velocity. One potential speculation for the performance degradation is a reduced firing rate of velocity-encoding neurons in the supraocular area. Another interpretation is that velocity-encoding neurons become less synchronized as the test progresses, or potentially both occur. Only a few participants experienced an increase in peak velocity, which could be described by velocity-encoding cells increasing their firing rates, firing more synchronously, or both. The underlying neurophysiological interpretation is speculative, as we do not have electrophysiological recordings from the participants in this study.

### 4.3. Differences Between Convergence and Divergence Systems

While not the primary aim of the study, our results are consistent with many other studies, in which divergence step responses have significantly lower peak velocities than convergence step responses (*p* < 0.001) [35,63,64]. These results can be explained by neurophysiological studies showing that distinct cells fire in response to convergence versus divergence stimuli [19,59,62]. Specifically, these studies on cellular firing rates show that the cellular population studies observed fewer divergence velocity-encoding cells than convergence velocity-encoding cells, which may explain in part why divergence responses have lower peak velocity than convergence responses.

The novelty of these results lies in comparing the endurance of the convergence and divergence systems. Our results support the idea that the endurance of the divergence oculomotor system was significantly different from that of the convergence oculomotor system (*p* < 0.03). The largest difference between convergence and divergence endurance metrics was 2% (1 participant), showing an increase in divergence peak velocity throughout the experiment, compared to 17% (eight participants), who showed an increase in convergence peak velocity as the test progressed. The differences are speculated to be potentially from the number of velocity-encoding cells between the convergence and divergence systems. Studies on macaques report more burst-velocity-encoding convergence cells than divergence cells [19,59,62]. Perhaps more convergence eye movement burst cells can improve synchronization with repetitive movements, thereby increasing peak velocity. Conversely, if fewer divergence velocity-encoding cells exist, improved synchronization within a smaller population of cells may not have as much influence on the overall eye-movement peak velocity. The results may also, in part, explain why patients typically do not have convergence and divergence dysfunction simultaneously.

### 4.4. Digital Eye Strain (DES) Implications

As our society evolves to become more dependent on digital electronic devices, the prevalence of DES increases, with studies noting that DES is present in up to 70% of the population [1,3,4] as does the number of studies on DES. Recent reviews support the idea that DES symptoms may be due to ocular conditions such as dry eye, refractive error, or binocular vision dysfunctions, as well as external variables such as ergonomics and lighting [65,66]. Given that the majority of the population experiences DES, more research is warranted. A 2025 review concludes that DES research would benefit from “… prospective longitudinal studies and well-designed [Randomized Control Trials] RCTs that integrate objective clinical measures to elucidate causal relationships and improve diagnostic and therapeutic frameworks” [67]. The vergence endurance test has the potential to serve as an objective clinical measure within RCTs.

### 4.5. Limitations and Future Direction

The goal of this research was to establish typical vergence oculomotor performance in individuals with normal binocular vision using a 15 min repetitive vergence eye-movement test that could be translated into a clinical setting. We could have continued the experiment to determine how many eye movements or how much time were needed to degrade performance in a larger group of neurologically normal participants with normal binocular vision, across a wider age range. However, our goal was to develop a tolerable test for use across various clinical populations. In addition, since some participants showed performance degradation in convergence (23% of participants) and divergence (29% of participants) responses, these results support the conclusion that the number of responses is sufficient to induce measurable fatigue for some. More importantly, the results are not ordinal (improvement, stable, degradation) but quantitative using a continuous scale, which is important for future clinical utility.

Future research includes testing this protocol across various patient populations to determine whether performance stability differs and, if so, to what extent the vergence endurance differs between groups. Furthermore, if differences between patients and neurologically normal patients are observed, then this test holds promise for therapeutic interventions to assess treatment effectiveness in future randomized clinical trials. During this study, most participants blinked only when the vergence demand was not changing, as instructed. Hence, blinks did not result in much data loss. Future studies on clinical populations may experience greater data loss due to blinks, which may result from decreased performance and fatigue.

The test duration was 15 min. For those with binocularly normal vision, we only needed to perform calibration at the beginning of the session because the head-mounted virtual reality headset was tightened in a comfortable position for the participant, and participants were asked not to touch the headset during the test. We instructed the participant to blink when the target was not moving; very few responses were omitted due to blinks. Dry eye was not reported by the participants. However, it is unclear whether headset drift, blinks, or dry eye will be a problem in patient populations. Beyond the scope of this study, a recent review paper has noted that variability among virtual reality headsets can be problematic [68] and should be assessed and accounted for if this test were used widely in clinical settings. Future studies would benefit from administering a visual symptom questionnaire before and after the vergence endurance test to determine whether the 15 min test affected symptoms. A post-test symptom survey was not conducted in this study, which is a limitation. In future research, if differences are observed among participants with brain injury, dysfunction, or disease and the results presented here for participants with binocularly normal vision, then research is suggested to establish normative values. Caution is warranted when using these findings, as systematic reviews have noted heterogeneity in normative values for oculomotor testing [69].

## 5. Conclusions

Convergence and divergence responses to symmetrical step stimuli during a repetitive 15 min test show that for those with binocularly normal vision, the peak velocity is maintained for the majority of participants. Some participants showed an increase in peak vergence velocity, while others exhibited a decrease as the test progressed. Significant differences were observed between the convergence and divergence systems. These results will serve as a comparison for vergence endurance, allowing the results reported here to be compared with those from individuals with acquired neurological dysfunctions, neurodegenerative disease, or those prone to digital eye strain. Furthermore, the vergence endurance test has the potential to assess therapeutic interventions that may improve oculomotor function.

## Figures and Tables

**Table 1:** Optometric sensory motor exam results and visual symptoms.

Optometric Sensory Motor Exam (*N* = 48):	Mean	95% CI	Standard Deviation
Spherical Refractive Error OD (D)	−0.6	−0.17 to −1.11	1.67
Spherical Refractive Error OS (D)	−0.8	−0.34 to −1.21	1.54
Dissociated Far (6 m) Heterophoria (Δ)	0.5 exo	0.3 exo to 0.7 exo	0.8 exo
Dissociated Near (40 cm) Heterophoria (Δ)	2.3 exo	1.3 exo to 3.3 exo	3.5 exo
Near Point of Convergence Break (cm)	3.2	2.9 to 3.6	1.2
Near Point of Convergence Recovery (cm)	4.4	4.1 to 4.8	1.3
Positive Fusional Vergence at Near (Δ)	27	24.5 to 29.5	8.8
Negative Fusion Vergence at Near (Δ)	14	13.0 to 14.9	3.3
Vergence Facility at Near (cpm)	17.6	16.3 to 18.8	4.5
Vergence Facility at Far (cpm)	15.8	14.5 to 17.2	4.8
Amplitude of Accommodation OD (D)	11.9	11.4 to 12.4	1.4
Amplitude of Accommodation OS (D)	12.0	11.5 to 12.6	1.8
Accommodative Facility (cpm)	12.4	10.5 to 14.2	6.5
**Visual Symptom Questionnaire:** via CISS (points)	9	7.63 to 10.33	4.8

D = diopter; Δ = prism diopter; OD = oculus dexter or right eye; OS = oculus sinister or left eye; cpm = cycles per minute; CISS = Convergence Insufficiency Symptom Survey; exo: exophoria; CI = confidence interval.

**Table 2:** Descriptives of slope of linear regression, peak velocity, and final amplitude for 48 participants.

Metric	Convergence (*N* = 48)	Divergence (*N* = 48)
Slope of Linear Regression		
Mean ± standard deviation	−0.03 ± 0.18	−0.08 ± 0.11
95% confidence interval	−0.08 to 0.03	−0.11 to −0.05
Peak Velocity (°/s)		
Mean ± standard deviation	20.0 ± 5.3	14.9 ± 4.6
95% confidence interval	18.5 to 21.6	13.6 to 16.2
Final Amplitude (°)		
Mean ± standard deviation	3.4 ± 0.4	3.3 ± 0.4
95% confidence interval	3.3 to 3.5	3.2 to 3.4

## Data Availability

The data presented in this study are available from the corresponding author upon request due to the proprietary nature of the data.

## Abbreviations

The following abbreviations are used in this manuscript:

BO	Base Out
BI	Base In
D	Diopter
95% CI	95% Confidence Interval
CISS	Convergence Insufficiency Symptom Survey
